# Metabolomic profiling of reactive persulfides and polysulfides in the aqueous and vitreous humors

**DOI:** 10.1038/srep41984

**Published:** 2017-02-07

**Authors:** Hiroshi Kunikata, Tomoaki Ida, Kota Sato, Naoko Aizawa, Tomohiro Sawa, Hiroshi Tawarayama, Namie Murayama, Shigemoto Fujii, Takaaki Akaike, Toru Nakazawa

**Affiliations:** 1 Department of Ophthalmology, Tohoku University Graduate School of Medicine, Sendai, Japan; 2 Department of Retinal Disease Control, Tohoku University Graduate School of Medicine, Sendai, Japan; 3 Department of Environmental Health Sciences and Molecular Toxicology, Tohoku University Graduate School of Medicine, Sendai, Japan; 4 Department of Microbiology, Graduate School of Medical Sciences, Kumamoto University, Kumamoto, Japan; 5 Department of Advanced Ophthalmic Medicine, Tohoku University Graduate School of Medicine, Sendai, Japan; 6 Department of Ophthalmic Imaging and Information Analytics, Tohoku University Graduate School of Medicine, Sendai, Japan

## Abstract

We investigate the metabolomic profile of reactive persulfides and polysulfides in the aqueous and vitreous humors. Eighteen eyes of 18 consecutive patients with diabetes mellitus (DM) and diabetic retinopathy underwent microincision vitrectomy combined with cataract surgery. Samples of the aqueous and vitreous humors were collected and underwent mass spectrometry-based metabolomic profiling of reactive persulfides and polysulfides (polysulfidomics). The effect of reactive polysulfide species on the viability of immortalized retinal cells (the RGC-5 cell line) under oxidative stress (induced with H_2_O_2_) was also evaluated with an Alamar Blue assay. The experiments showed that cysteine persulfides (CysSSH), oxidized glutathione trisulfide (GSSSG) and cystine were elevated in the aqueous humor, and CysSSH, Cys, and cystine were elevated in the vitreous. Furthermore, GSSSG, cystine, and CysSSH levels were correlated in the aqueous and vitreous humors. A comparison, in DM and control subjects, of plasma levels of reactive persulfides and polysulfides showed that they did not differ. *In vitro* findings revealed that reactive polysulfide species increased cell viability under oxidative stress. Thus, various reactive persulfides and polysulfides appear to be present in the eye, and some reactive sulfide species, which have a protective effect against oxidative stress, are upregulated in the aqueous and vitreous humors of DM eyes.

Diabetic retinopathy (DR) is a common retinal disease in patients with diabetes mellitus (DM) that can result in neovascularization, fibrovascular proliferation, tractional detachment, and vitreous hemorrhage, and often leads to severe visual disturbance. There are over 350 million people with DM worldwide^1^, approximately one third of whom currently have or will develop DR. Thus, DM has become a major cause of visual disability among working-aged adults in developing and developed countries^2^. DR is particularly serious after it progresses to proliferative DR (PDR) or diabetic macular edema (DME), because treatment then becomes difficult even for experienced ophthalmologists^3^,^4^,^5^. Thus, despite recent progress, there is still a need for improved treatments, which will require a better understanding of the underlying mechanisms of DR. Experimental models suggest that oxidative stress and consequent retinal cell death are important^6^,^7^,^8^. Here, in order to reveal more details of the pathomechanism of DR, we examined clinically harvested intraocular samples from patients with DR, identified potentially important metabolites, and determined the significance of these metabolites in retinal cells *in vitro*.

One of the key goals of this study was to better understand the impact of oxidative stress in DR. Oxidative stress is mainly induced by reactive oxygen species (ROS), which cause damage to cells by attacking proteins, lipids and nucleic acids. ROS can become especially elevated in infectious or inflammatory diseases, malignant tumors, after ischemia/reperfusion injury, and in metabolic disorders such as DM. Recently, the level of ROS was reported to be closely associated with signal transduction. ROS and nitric oxide (NO) generate 8-nitroguanosine 3′,5′-cyclic monophosphate (8-nitro-cGMP), which is involved in signal transduction as a secondary messenger of ROS signals^9^,^10^. Thus, ROS can cause specific changes by impairing homeostasis of the redox signal, as well as cause non-specific changes that are level-dependent and toxicity-induced. Furthermore, reactive persulfides/polysulfides, including cysteine persulfides (CysSSH), have been reported to form endogenously, from both small molecule species and from proteins, in high amounts in mammalian cells and tissues, including human plasma^11^. These persulfide/polysulfide molecules have also been found to have potent antioxidant and redox signaling functions^11^. This finding has led to better understanding of the regulatory mechanism for ROS signals and speculation that DM, DR and retinal cell degeneration are closely associated with ROS-induced hyperglycemia and oxidative stress^8^,^12^,^13^,^14^. Nevertheless, the pathogenesis of DR is complex, and many of its aspects remain unclear. In particular, the metabolomic profile of reactive persulfides and polysulfides in the aqueous humor and vitreous remains unknown.

In this study, therefore, we set out to determine whether reactive sulfide species exist in the intraocular cavity in humans, and if they exist, what effect they have on retinal cells. To achieve this goal, we first used a liquid chromatography electrospray ionization tandem mass spectrometry (LC-ESI-MS/MS)-based metabolomic method (polysulfidomics) to profile low-molecular weight reactive polysulfides and their related molecular species in the aqueous humor and vitreous of DM patients with DR, and then examined the effect of the reactive sulfide species we identified on the viability of cultured retinal cells under oxidative stress.

## Results

Eighteen eyes of 18 patients (9 men and 9 women) with PDR were included in this study. The ages of the patients ranged from 27 to 82 years with a mean of 57.7 ± 16.1 years. Twenty-two patients with ERM, MH, or cataract without retinal disease served as controls. Table 1 shows the characteristics of the DM and normal subjects. The two groups had no differences in sex, body weight, or total cholesterol, but had significant differences in age, glycosylated hemoglobin, glucose, urea nitrogen, and creatinine (Table 1). Additionally, the age of the male subjects differed significantly between the DM and control groups (*P* = 0.004), while the age of the female subjects was similar. No severe adverse events, such as endophthalmitis, or any systemic side effects were observed in this study.

**Figure 1:**
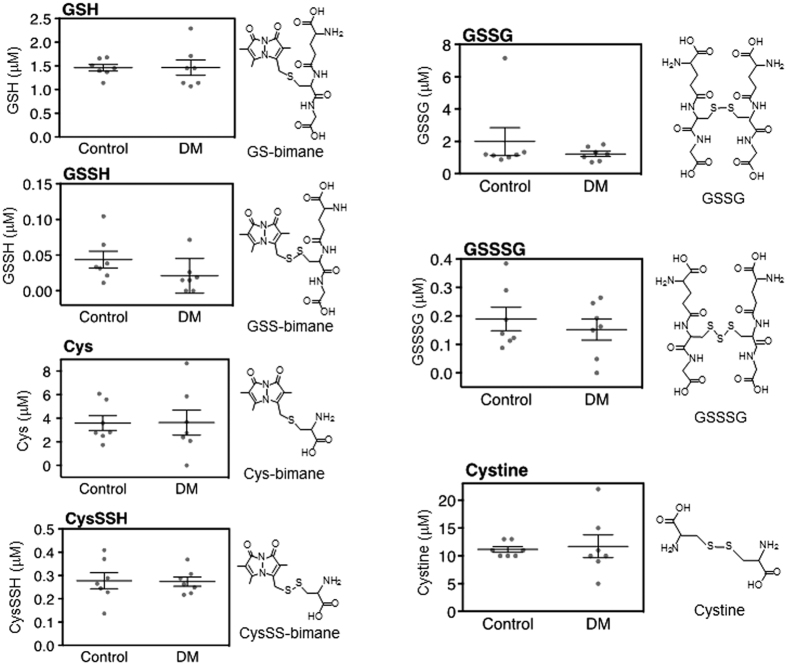
Metabolomic profile of the plasma of diabetes mellitus (DM) patients with diabetic retinopathy and normal controls. GSH, GSSH, Cys, CysSSH, GSSG, GSSSH and cystine were all detected in the plasma of both DM patients (*n* = 7) and controls (*n* = 7). There were no significant differences in the concentrations of these reactive sulfide species in the DM patients and controls.

**Figure 2:**
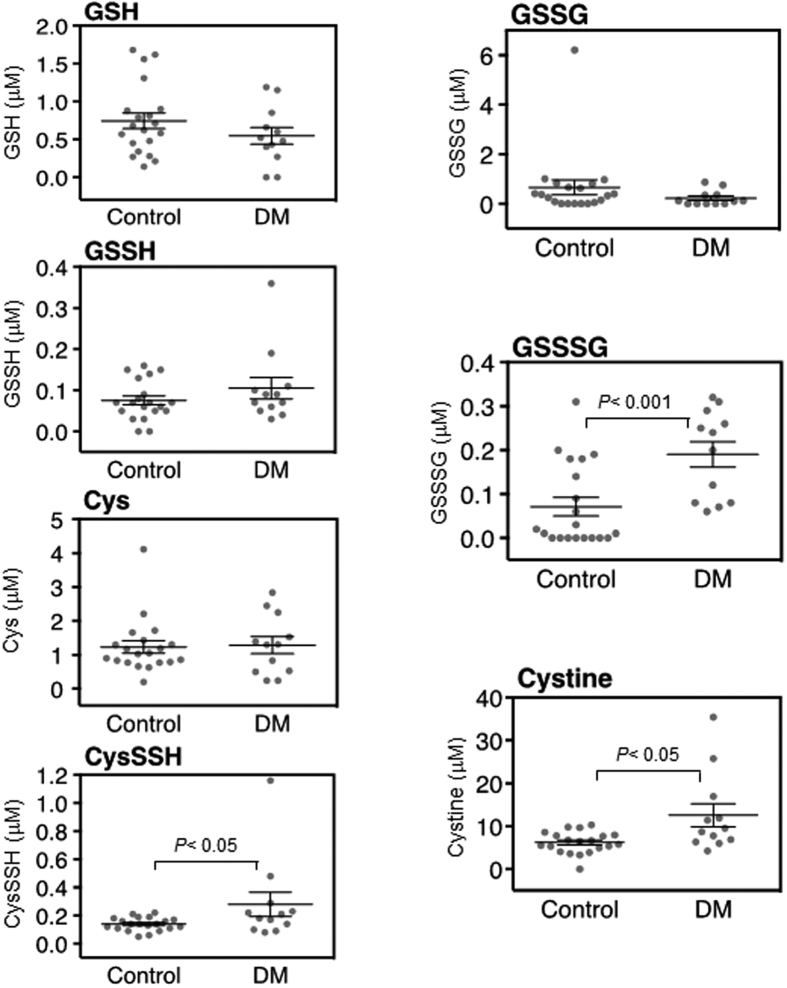
Metabolomic profile of the aqueous humor in diabetes mellitus (DM) patients with diabetic retinopathy and normal controls. GSH, GSSH, Cys, CysSSH, GSSG, GSSSG and cystine were all detected in the aqueous humor of both DM patients (*n* = 12) and controls (*n* = 20). There were no significant differences in the concentrations of GSH, GSSH, Cys, or GSSG in the DM patients and controls. CysSSH, GSSSG and cystine had higher concentrations in the aqueous humor of the DM patients than the controls (*P* < 0.05, *P* < 0.001, and *P* < 0.05, respectively).

**Figure 3:**
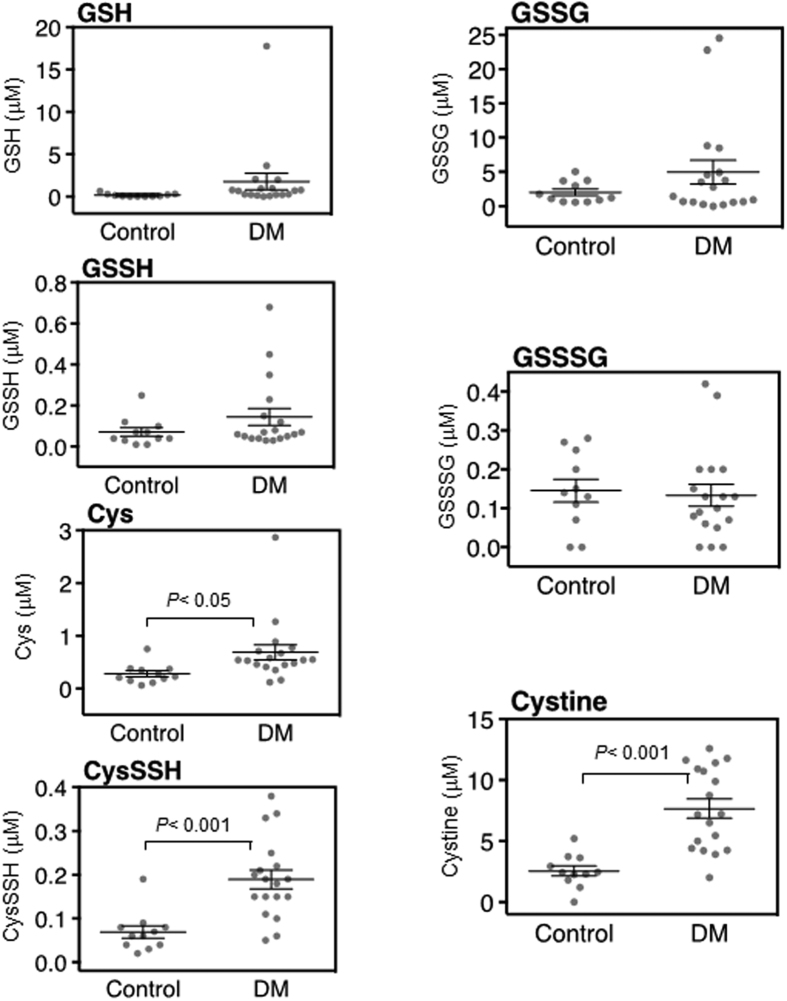
Metabolomic profile of the vitreous humor in diabetes mellitus (DM) patients with diabetic retinopathy and normal controls. GSH, GSSH, Cys, CysSSH, GSSG, GSSSG and cystine were all detected in the vitreous of both DM patients (*n* = 18) and controls (*n* = 11). There were no significant differences in the concentrations of GSH, GSSH, GSSG or GSSSG in DM patients and controls. Cys, CysSSH and cystine had higher concentrations in the vitreous humor of DM patients than controls (*P* < 0.05, *P* < 0.001, and *P* < 0.001, respectively).

Our LC-ESI-MS/MS-based polysulfidomics for sulfhydryl and reactive persulfides/polysulfides can identify 31 sulfide derivatives, including glutathione (GSH), glutathione hydropersulfide (GSSH), Cys, CysSSH, glutathione disulfide (GSSG), glutathione trisulfide (GSSSG) and cystine, in any type of clinical specimen. Among these 31 derivatives, we found that four reactive persulfides/polysulfides had significantly different concentrations in the aqueous and vitreous humors of the DM and control patients. In the plasma, none of these four reactive persulfides/polysulfides differed significantly (Fig. 1 and Supplementary Table S1). In the aqueous, the DM subjects had higher levels of CysSSH, GSSSG and cystine (*P* < 0.05, *P* < 0.001, and *P* < 0.05, respectively; Fig. 2, Supplementary Figure S1 and Supplementary Table S1). In the vitreous, the DM subjects had higher levels of Cys, CysSSH and cystine (*P* < 0.05, *P* < 0.001, and *P* < 0.001, respectively; Fig. 3, Supplementary Figure S2 and Supplementary Table S1). All other sulfide species in the analysis had statistically similar levels in the aqueous and vitreous humors of the DM and control eyes.

**Figure 4:**
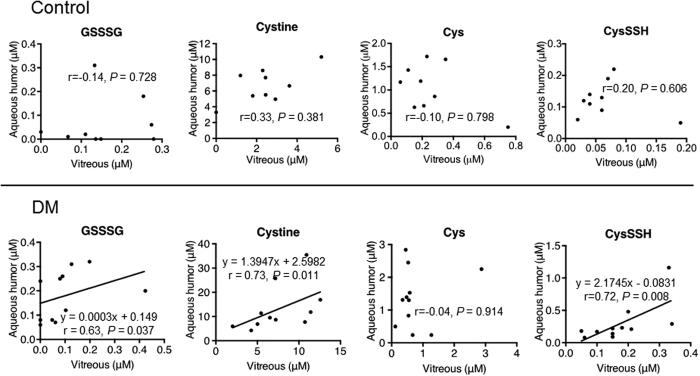
Relationship between metabolomic profiles of the aqueous and vitreous humor in diabetes mellitus (DM) patients with diabetic retinopathy and normal controls. In the controls (*n* = 9), the aqueous and vitreous concentrations of GSSSG, cystine, Cys, and CysSSH were not correlated (upper). In the DM patients (*n* = 11), the aqueous and vitreous concentrations of GSSSG, cystine and CysSSH were correlated (r = 0.63, *P* < 0.05; r = 0.73, *P* = 0.01; r = 0.72, *P* = 0.01), although the aqueous and vitreous concentrations of Cys were not (lower).

In the controls, the aqueous and vitreous concentrations of GSSSG, cystine, Cys, and CysSSH were not correlated (Fig. 4 upper). In the DM patients, the aqueous and vitreous concentrations of GSSSG, cystine and CysSSH were correlated (r = 0.63, *P* < 0.05; r = 0.73, *P* = 0.01; r = 0.72, *P* = 0.01), although the aqueous and vitreous concentrations of Cys were not (Fig. 4 lower).

**Figure 5:**
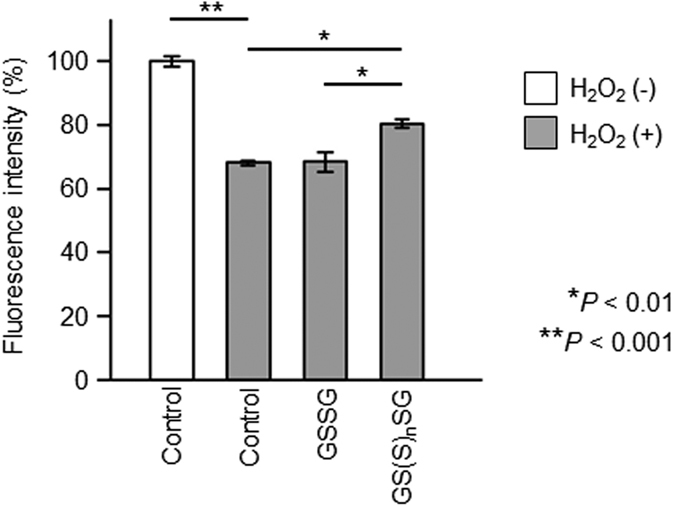
Cell viability after oxidative stress exposure with or without reactive sulfide species. Exposing glutathione-depleted RGC-5 cells to high levels of hydrogen peroxide (H_2_O_2_; 100 μM) resulted in a remarkable reduction in cell viability (H_2_O_2_(−): 100.0 ± 3.8%; H_2_O_2_(+): 68.1 ± 1.5%; n = 4; **P < 0.001). However, this H_2_O_2_-mediated negative effect on cell survival was significantly attenuated by GS(S)_n_SG (80.4 ± 2.5%; n = 4; *P < 0.01), although not by GSSG (68.4 ± 5.6%; n = 4), at a final concentration of 25 μg/ml.

To gain insight into the pathological role of increased GSSSG in the aqueous humor of patients with DR, we next investigated the effect of various glutathione polysulfides (GS(S)_n_SG) on the survival of retinal cells (specifically, an immortalized line of RGC-5 cells) under conditions of high oxidative stress *in vitro*. The RGC-5 cells were treated exogenously with a mixture of three different GS(S)_n_SG: GSSSG (84%), GSSSSG (15%), and GSSSSSG (1%) (i.e., excluding GSSG) in the presence of hydrogen peroxide (H_2_O_2_) after 15 hours of incubation with a glutathione synthesis inhibitor, buthionine sulphoximine (BSO) (Supplementary Figure S3). This revealed that BSO treatment drastically reduced endogenous glutathiones in RGC-5 cells, a result that is consistent with previous findings^15^, and also led to a higher susceptibility of the cells to H_2_O_2_-mediated oxidative stress (Supplementary Figure S4). Furthermore, exposure of glutathione-depleted RGC-5 cells to excessive H_2_O_2_ (100 μM) resulted in a remarkable reduction in cell viability (H_2_O_2_(−): 100.0 ± 3.8%; H_2_O_2_(+): 68.1 ± 1.5%; n = 4; *P* < 0.001; Fig. 5). Moreover, the impact of H_2_O_2_ on cell survival was significantly attenuated by exogenous GS(S)_n_SG (80.4 ± 2.5%; n = 4; *P* < 0.01 vs. control), but not by GSSG (68.4 ± 5.6%; n = 4), at a final concentration of 25 μg/ml (Fig. 5). Finally, we found that the exogenous glutathione derivatives at these concentrations had no cytotoxic effects on the glutathione-depleted RGC-5 cells (Supplementary Figure S4).

## Discussion

We set out to investigate the metabolomic profile of reactive persulfides and polysulfides in the aqueous and vitreous humors. Our first step was to profile, with an MS-based metabolomic method, reactive persulfides and polysulfides in the aqueous humor, vitreous humor, and plasma of patients with PDR and control subjects. We found that the two study groups had no significant differences in reactive persulfides or polysulfides in the plasma, but that CysSSH, GSSSG, and cystine were higher in the aqueous humor of the DM than the control eyes, and that CysSSH, Cys, and cystine were higher in the vitreous humor of the DM than the control eyes. Furthermore, the DM eyes showed a significant correlation between the aqueous and vitreous levels of GSSSG, cystine and CysSSH. The second step of our investigation was to perform an *in vitro* study of retinal cell viability in the presence of reactive persulfides and polysulfides. After confirming that H_2_O_2_ exposure lowered the survival rate in an RGC-5 culture, we tested the effect of combined H_2_O_2_ exposure/GSSG treatment on cell survival. We found that the RGC-5 survival rate with GSSG and H_2_O_2_ exposure was similar to the survival rate without GSSG. However, the survival rate was significantly higher when H_2_O_2_ exposure was combined with treatment with a glutathione polysulfide species, GS(S)_n_SG.

In many fields of medicine, metabolomics has become a common way of identifying biomarkers of a wide variety of diseases. Like genomics and proteomics, which measure the genome and proteome, respectively, metabolomics measures the metabolome, which represents the metabolic consequences of protein activity, and, ultimately, genetic expression. Thus, the total physiological environment of the living cell at a particular moment, including the genetic, protein, and metabolic levels, is now open to research. Metabolomics can provide new information on processes or pathways involved in the pathogenesis of a targeted disease, or in the response to a therapy. Recent research has shown that the metabolomics based on proton nuclear magnetic resonance (1H-NMR) is an accurate, noninvasive and rapid way to diagnose coronary heart disease and its severity^16^. Another large-scale, exploratory study used 1H-NMR to determine metabolic phenotypes in urine samples. That study showed that metabolomics can advance our understanding of the pathogenesis of cardiovascular disease and reveal related biomarkers^17^. Additionally, LC-MS analysis of metabolites in cell lines and neuroendocrine tumors of the prostate can identify human neuroendocrine cancers with poor prognoses^18^.

In ophthalmology, metabolomics is an emerging and potentially powerful tool^19^, but remains relatively rare. Its potential uses include characterizing healthy biofluids, studying tissue metabolism, elucidating disease mechanisms, identifying disease biomarkers and risk factors, and accelerating the development of new therapies for ocular disease^19^. A special challenge in applying metabolomics techniques in ophthalmology is the difficulty of obtaining intraocular fluids or tissues, in comparison with blood or urine samples. However, intraocular samples are available when patients undergo cataract or vitreous surgeries. Indeed, we believe that the most promising source of biomarkers of retinal diseases is the vitreous humor, because the vitreous humor is particularly easy to extract during surgery and because it is in anatomical contact with the retina. The first successful application of a vitreous-metabolomic analysis was in differentiating types of vitreoretinal diseases, including chronic non-infectious uveitis and lens-induced uveitis^20^. This was followed by a vitreous-metabolomic study of the differences between PDR patients and non-diabetic controls^21^, and a study of the differences between patients with rhegmatogenous retinal detachment and proliferative vitreoretinopathy^22^. These studies suggest that metabolomic profiling of vitreous samples may become increasingly useful in guiding the diagnosis of vitreoretinal disease, forming prognoses, and assessing the response to treatment. This technique is especially promising for inflammatory diseases, because ocular inflammatory disease can have tissue-specific as well as systemic components^19^,^22^,^23^.

The metabolomic profile of reactive persulfides and polysulfides in human fluids and tissue is difficult to assess, and thus reports are still very rare^11^. Furthermore, different types of metabolites, such as lipids, amino acids, and organic acids, have different metabolomic profiles, and there is no established or convenient methodology to measure the profile of the various polysulfide species (e.g., RSS_n_H and RS(S)_n_R; R = Cys and GSH, *n* ≥ 1). A previous report used an MS-based method to identify biologically relevant synthetic polysulfide compounds, and examined the endogenous formation of polysulfide-related products and metabolites^11^. In that report, oxidized thiols were reacted with H_2_S to generate persulfides and derived species and to synthesize various polysulfides and/or hydropolysulfides (RS(S)_n_H). The results confirmed a variety of reactive sulfide species are present in the plasma of both human beings and mice^11^. Other research has used metabolomic profiling of blood samples to identify retinal diseases. One report used LC-MS to identify distinct metabolomic profiles in the plasma of patients that were associated with anterior uveitis^24^, and another identified profiles associated with neovascular age-related macular degeneration^25^. A third study used metabolomic analysis of lipids to show that the lipid composition of red blood cells may be a good mirror of fatty acids in the retina and the optic nerve, a finding that promises to allow the creation of an index of ocular disease risk^26^. Finally, a gas chromatography (GC)-MS-based study of the amino acid metabolome in the serum, including pyruvic acids and L-aspartic acid, was able to differentiate DM patients into pre-clinical, non-proliferative and PDR groups^27^. However, this analysis was not LC-MS-based, and did not identify a distinct metabolomic profile of reactive polysulfide species in the plasma of the PDR group, although it did identify such a profile in the intraocular fluid of the PDR group. It is unclear why, but it may be that excess reactive polysulfide species produced by intraocular tissues are not transported to the extraocular circulation, even when the blood-retinal barrier has been impaired by diabetes. It may also be that the polysulfide species are too diffuse to be measured in the systemic circulation after transportation.

One of the most interesting findings of the current study was the intraocular upregulation of reactive persulfides and polysulfides, including CysSSH, in the DM group. The DM group also showed a significant correlation between the aqueous and vitreous levels of GSSSG, cystine and CysSSH, a finding that should allow clinicians to infer the vitreous levels of these compounds based on their aqueous levels. This would be a great benefit, as aqueous levels can easily be sampled in an outpatient setting, whereas obtaining vitreous samples requires invasive surgery. Reactive sulfide species have been reported to be generated endogenously in living animals, and to scavenge ROS, thus acting as powerful antioxidants^11^. CysSSH is the result of the combination of Cys, an amino acid, with extensive quantities of sulfide. CysSSH is synthesized from cystine by cystathionine β-synthase and cystathionine γ-lyase, and may contribute, in turn, to high levels of GSSH and other CysSSH derivatives in various organs in mice. Compared with GSH and H_2_S, CysSSH derivatives are superior nucleophiles and reductants, capable of regulating electrophilic cell signaling mediated by 8-nitro-cGMP. 8-Nitro-cGMP seems to regulate the redox-sensor signaling protein Keap1, a potent repressor of Nrf2-dependent transcription, via S-guanylation of the highly nucleophilic cysteine sulfhydryls of Keap1^10^, and the metabolism of 8-nitro-cGMP may thus also be valuable in the development of new approaches to the diagnosis and treatment of diseases related to oxidative stress and redox metabolism^28^. These findings suggest that reactive Cys persulfides and polysulfidation have critical regulatory functions in redox cell signaling, and can prevent specific changes induced by impaired redox homeostasis. It is well known that sulfur-rich foods, such as garlic, promote good health and have disease-preventive effects^29^,^30^,^31^. Interestingly, there is a long-standing belief in Japan that sulfur-rich natural hot springs can aid in injury recovery. Scientific evidence for a mechanism underlying such an effect has never been put forth, but we consider that the role of reactive sulfide species in counteracting the effects of ROS may explain this historical belief, which may in fact be more than a mere superstition. Nevertheless, it remains unclear why intraocular reactive sulfide species are upregulated in DM, as the current study found. One possible explanation might be that this upregulation compensates for excess oxidative stress in eyes with DM. The *in vitro* experiment in this study, which showed a protective effect against oxidative stress in the retinal cells, supports this hypothesis, although the concentration of reactive sulfide species in the experimental study was higher than in the intraocular samples. Thus, this explanation remains to be confirmed in a future, animal model-based *in vivo* study.

The limitations of this study included clinical differences in the patient background of the groups, particularly age (most particularly the age of the male patients), and a small sample size. Thus, differences in the serum levels of intraocular metabolites between the groups might have been affected by sex and age. Moreover, we could not study changes in the retinal metabolome, which is very important for the understanding of DM, due to ethical considerations. Additionally, the *in vitro* study used only mixed reactive polysulfide species (GS(S)_n_SG), i.e. GSSSG, GSSSSG, and GSSSSSG, due to the enormous cost and difficulty of purifying samples of each reactive persulfide and polysulfide, including CysSSH. Nevertheless, taken together with our previous study demonstrating that GS(S)_n_SG is endogenously produced in various tissues^11^, the current findings suggest that GS(S)_n_SG might exert a prominent physiological function as an antioxidant, especially in comparison with GSSG. Furthermore, in this study, patient selection was very careful, intraocular fluid was harvested without touching any intraocular tissues, and LC-ESI-MS/MS was used, which is a very sensitive metabolomic technique in small samples. Therefore, we believe that our main finding, i.e., that various reactive sulfide species are upregulated in eyes with DM, can be considered reliable and will support future efforts to better understand the pathology and pathomechanisms of DM.

In conclusion, this is the first report to use metabolomics to profile reactive persulfides and polysulfides in the human intraocular cavity and plasma. We found that reactive sulfide species did not differ in the plasma of DM patients and non-diabetic controls, but that CysSSH, GSSSG, and cystine were higher in the aqueous humor of DM eyes than controls, and that CysSSH, Cys, and cystine were higher in the vitreous humor of DM eyes than controls. Furthermore, the aqueous and vitreous humor levels of GSSSG, cystine, and CysSSH were correlated in the DM patients, but not in the controls. Finally, an *in vitro* study of cell viability under oxidative stress showed that cell survival was higher in the presence of reactive sulfide species than in their absence. Taken together with our previous work^11^, the current results showed that GS(S)_n_SG, a type of polysulfides species, are less common in harvested human tissue than GSSG, but might have a stronger anti-oxidative effect. Furthermore, the fact that reactive sulfide species, which might protect retinal cells from oxidative stress damage, are upregulated in eyes with diabetic retinopathy leads us to believe that these reactive sulfide species may be promising target molecules for therapy to treat diabetic retinopathy. Further investigation of diabetic retinopathy is needed to evaluate the relationship between these reactive sulfide species and clinical findings, to understand the metabolism of the disease, to elucidate its pathogenesis, and to identify new biomarkers.

## Materials and Methods

### Setting and design

This study comprised an institutional, retrospective, non-randomized, interventional case series and an experimental *in vitro* investigation using cultured cells.

### Patients

Subjects were recruited from patients referred to the Surgical Retinal Service of Tohoku University Hospital. Surgical intervention and follow-up were both performed at this clinic. All patients had PDR and were studied before combined microincision vitrectomy surgery (MIVS)/cataract surgery was performed. The inclusion criterion was clinically detectable PDR. The exclusion criteria were prior vitreous surgery, the intravitreal injection of triamcinolone acetonide (IVTA) or anti-vascular endothelial growth factor (VEGF), and the presence of ocular inflammation or vitreoretinal and optic nerve diseases.

Informed consent was obtained from all patients for both treatment and participation in this retrospective study (University Hospital Medical Information Network; UMIN Study ID N.: UMIN000021589). The Institutional Review Board of Tohoku University Graduate School of Medicine approved the study methodology. The research was conducted according to the provisions of the Declaration of Helsinki, 1995 (as revised in Edinburgh, 2000).

### Intervention and sample collection

All PDR patients underwent MIVS combined with cataract surgery using Constellation instruments (Alcon Laboratories; Fort Worth, Texas, USA). Before surgery, blood samples were obtained with heparin-containing tubes (Venoject II blood collection tube, Terumo Corporation, Tokyo, Japan) via an intravenous drip infusion system, from which plasma was separated by centrifuge. Hemoglobin A1c (HbA_1c_), glucose, total cholesterol, urea nitrogen, and creatinine were measured with automated standardized laboratory techniques. Aqueous humor samples were collected before the start of cataract surgery. Following this, vitreous samples were obtained during MIVS, which was performed with 25-gauge instruments. Vitreous hemorrhage, the fibrovascular membrane and the internal limiting membrane were removed and fluid air exchange was performed, if necessary. Control subjects were recruited from patients undergoing combined MIVS/cataract surgery for epiretinal membrane (ERM) or macular hole (MH), or for cataract surgery for senile cataract. Samples of the aqueous and vitreous humors were obtained in the same fashion as the patients undergoing surgery for PDR. The samples of aqueous and vitreous humor each comprised about 100 μl. Special care was taken to avoid touching intraocular tissues, i.e., the cornea, the iris, and the lens, and to prevent mixing intraocular samples with other fluids.

### Measurement of reactive polysulfide species

After collection in sterile tubes, the samples were immediately frozen at −80 °C. The control samples of the aqueous humor, obtained from eyes undergoing vitreous surgery for ERM or MH, were also immediately frozen. The samples were analyzed using the LC-ESI-MS/MS-based metabolomic method (polysulfidomics) as described below. A total volume of 50 μl from each sample was used for the assay.

### LC-ESI-MS/MS analysis for polysulfidomics

Sulfide-related metabolite profiling was conducted with the LC-ESI-MS/MS approach described in our previous paper^11^. In brief, the aqueous and vitreous humors, as well as the plasma, were treated with the addition of a 5 vol excess of a methanol solution containing 5 mM monobromobimane (Sigma-Aldrich, St. Luis, MO) and were incubated at 37 °C for 15 min. After centrifugation, aliquots of the supernatants of the monobromobimane-treated samples were diluted 10–100 times with distilled water containing known amounts of isotope-labeled internal standards, which were synthesized as in our previous work^11^, for quantitative analysis. We used an Agilent 6430 Triple Quadrupole LC-mass spectrometer (Agilent Technologies, Santa Clara, CA) to perform LC-ESI-MS/MS. Ionization was achieved with electrospray in the positive mode, and polysulfide derivatives were identified and quantified by means of multiple reaction monitoring. Various per/polysulfide derivatives were identified and quantified by means of multiple reaction monitoring for each derivative. The parameters for multiple reaction monitoring, as well as the LC-ESI-MS/MS conditions, are described in our earlier report^11^.

### Cell culture

Immortalized retinal ganglion cells (RGC-5) were cultured in Dulbecco’s Modified Eagle Medium (DMEM; Sigma-Aldrich, St. Luis, MO), supplemented with 10% fetal bovine serum (FBS; Biological Industries, Cromwell, CT), in a CO_2_ incubator at 37 °C. Cells were subcultured upon reaching 80% confluency using 0.25% trypsin-EDTA solution (GIBCO BRL, Palo Alto, CA).

### Cell viability assay

RGC-5 cells were cultured for 15 hours in a DMEM/FBS medium containing BSO(500 μM; Wako Pure Chemical, Osaka, Japan) to deplete endogenous glutathiones, and then exogenously treated with glutathione-disulfide (25 μg/ml; Wako Pure Chemical) or -persulfide (25 μg/ml)^11^ for 6 hours in the presence of glutathione reductase and its coenzyme nicotinamide adenine dinucleotide phosphate (NADPH). These components were supplied as part of the GSSG/GSH Quantification Kit (Dojindo Laboratories, Kumamoto, Japan). Hydrogen peroxide was added to the cultures at a final concentration of 100 μM to trigger oxidative stress and cell death in the RGC-5 cells. After a 90-minute treatment with hydrogen peroxide, the viability of the cells was evaluated with an Alamar Blue assay (Invitrogen, Carlsbad, CA). For this assay, 1 part of Alamar Blue reagent was added to 9 parts of the culture, and fluorescence was then measured, at an excitation wavelength of 560 nm and an emission wavelength of 590 nm, with a fluorescence microplate reader (SpectraMax Gemini; Molecular Devices LLC, Sunnyvale, CA). The surviving RGC-5 cells were measured as a percentage of surviving cells in a negative control, which comprised cells treated with PBS and without hydrogen peroxide.

### Preparation of GS(S)_n_SG

GS(S)_n_SG was prepared as in our previous work^11^ with slight modifications. In brief, GSH (20 mM) was reacted with 20 mM NaHS in the presence of I_2_ (20 mM) in 20 mM Tris-HCl buffer (pH 7.4) at room temperature for 15 min. The reaction mixture was purified with 35 ml of Sep-Pak Vac Cartridge (Waters, Milford, MA). The GS(S)_n_SG was washed with 200 ml of 0.1% formate and eluted with 60% methanol containing 0.1% formate. The eluted samples were dried *in vacuo* and then re-dissolved in distilled water.

### Measurement of oxidative stress damage and the protective effect of reactive polysulfide species

The effect of glutathione polysulfides (GS(S)_n_SG) on cells under oxidative stress was investigated with RGC-5 cells, which are an immortalized line of mouse RGCs^32^. The RGCs were cultured in a medium containing GSSG or GS(S)_n_SG in the presence of glutathione reductase, which converts the oxidized form of glutathiones into a reduced form capable of scavenging ROS. Endogenous glutathiones were depleted via treatment with BSO.

### Main outcome measure

The main outcome was a profile of reactive persulfides and polysulfides in the aqueous and vitreous humor of patients with PDR. These reactive sulfide species were compared in the PDR eyes and control eyes. The relationship between the concentrations of various reactive sulfide species in the aqueous and vitreous humors was also determined. Finally, the viability of cells under oxidative stress in the presence or absence of various reactive sulfide species was evaluated with an Alamar Blue assay.

### Statistical analyses

All statistical analyses were performed with JMP software (Pro version 10.0.2, SAS Institute Japan Inc., Tokyo, Japan) and Prism ver. 6.0 (GraphPad Software, San Diego, CA). The data are presented as mean ± standard error of the mean. An unpaired t-test was used to compare differences in background characteristics between groups. The chi-square test was used for frequency data on sex. The significance of differences in the concentration of reactive polysulfide species in the aqueous and vitreous humor of the DM and control eyes was assessed with the Student’s t-test. Spearman’s coefficient of correlation by rank was used to determine the correlation between the aqueous and vitreous humor levels of the reactive polysulfide species. The significance of differences in cell viability under oxidative stress with and without reactive polysulfide species was evaluated with the Tukey-Kramer test. A *P* value of less than 0.05 was considered to be statistically significant.

## Additional Information

**How to cite this article:** Kunikata, H. *et al*. Metabolomic profiling of reactive persulfides and polysulfides in the aqueous and vitreous humors. *Sci. Rep.* **7**, 41984; doi: 10.1038/srep41984 (2017).

**Publisher's note:** Springer Nature remains neutral with regard to jurisdictional claims in published maps and institutional affiliations.

## Supplementary Material

Supplementary Information

## Figures and Tables

**Table 1:** Characteristics of diabetes mellitus (DM) patients with diabetic retinopathy and normal controls.

N	DM	Control	*P* value
	18	22	
Age (years)	57.7 ± 16.1	71.0 ± 13.4	0.007
Sex (M:F)	9:9	9:13	0.565^a^
BW (kg)	58.6 ± 13.6	60.3 ± 9.9	0.652
HbA1c (%)	7.5 ± 2.0	5.9 ± 0.8	0.004
Glu (mg/dl)	175.4 ± 74.8	101.0 ± 14.9	<0.001
T-chol (mg/dl)	182.1 ± 45.1	187.3 ± 39.4	0.701
UN (mg/dl)	19.5 ± 7.1	14.9 ± 2.9	0.009
Cre (mg/dl)	1.1 ± 0.6	0.8 ± 0.2	0.040

BW: body weight, cre: creatinine, DM: diabetes mellitus, glu: glucose.

HbA1c: glycosylated hemoglobin, T-chol: total cholesterol, UN: urea nitrogen.

Unmarked *P* value: unpaired t test, a: Chi-square test.
