# Supplementary material for: Metabolomic profiling of reactive persulfides and polysulfides in the aqueous and vitreous humors
Source: Sci Rep. 2017 Feb 7;7:41984. doi: 10.1038/srep41984 (PMC5294455; doi:10.1038/srep41984)
Supplement: Supplementary Information [file srep41984-s1.pdf]

Supplementary information

Metabolomic profiling of reactive persulfides and polysulfides in the aqueous and vitreous humors

Hiroshi Kunikata[1,2]* MD, PhD, Tomoaki Ida[3] PhD, Kota Sato[1] PhD, Naoko Aizawa[1] MD, PhD, Tomohiro Sawa[4] PhD, Hiroshi Tawarayama[2] PhD, Namie Murayama[1], Shigemoto Fujii[3] PhD, Takaaki Akaike[3] MD, PhD, and Toru Nakazawa[1, 2, 5, 6] MD, PhD

[1]Department of Ophthalmology, Tohoku University Graduate School of Medicine, Sendai, Japan

[2]Department of Retinal Disease Control, Tohoku University Graduate School of Medicine, Sendai, Japan

[3]Department of Environmental Health Sciences and Molecular Toxicology, Tohoku University Graduate School of Medicine, Sendai, Japan.

[4]Department of Microbiology, Graduate School of Medical Sciences, Kumamoto University, Kumamoto, Japan

[5]Department of Advanced Ophthalmic Medicine, Tohoku University Graduate School of Medicine, Sendai, Japan

[6]Department of Ophthalmic Imaging and Information Analytics, Tohoku University Graduate School of Medicine, Sendai, Japan

Corresponding author: Hiroshi Kunikata MD, PhD

Department of Ophthalmology, Tohoku University Graduate School of Medicine, 1-1 Seiryo-machi, Aoba-ku, Sendai 980-8574, Japan

E-mail: kunikata@oph.med.tohoku.ac.jp

TEL: +81-22-717-7294, FAX: +81-22-717-7298

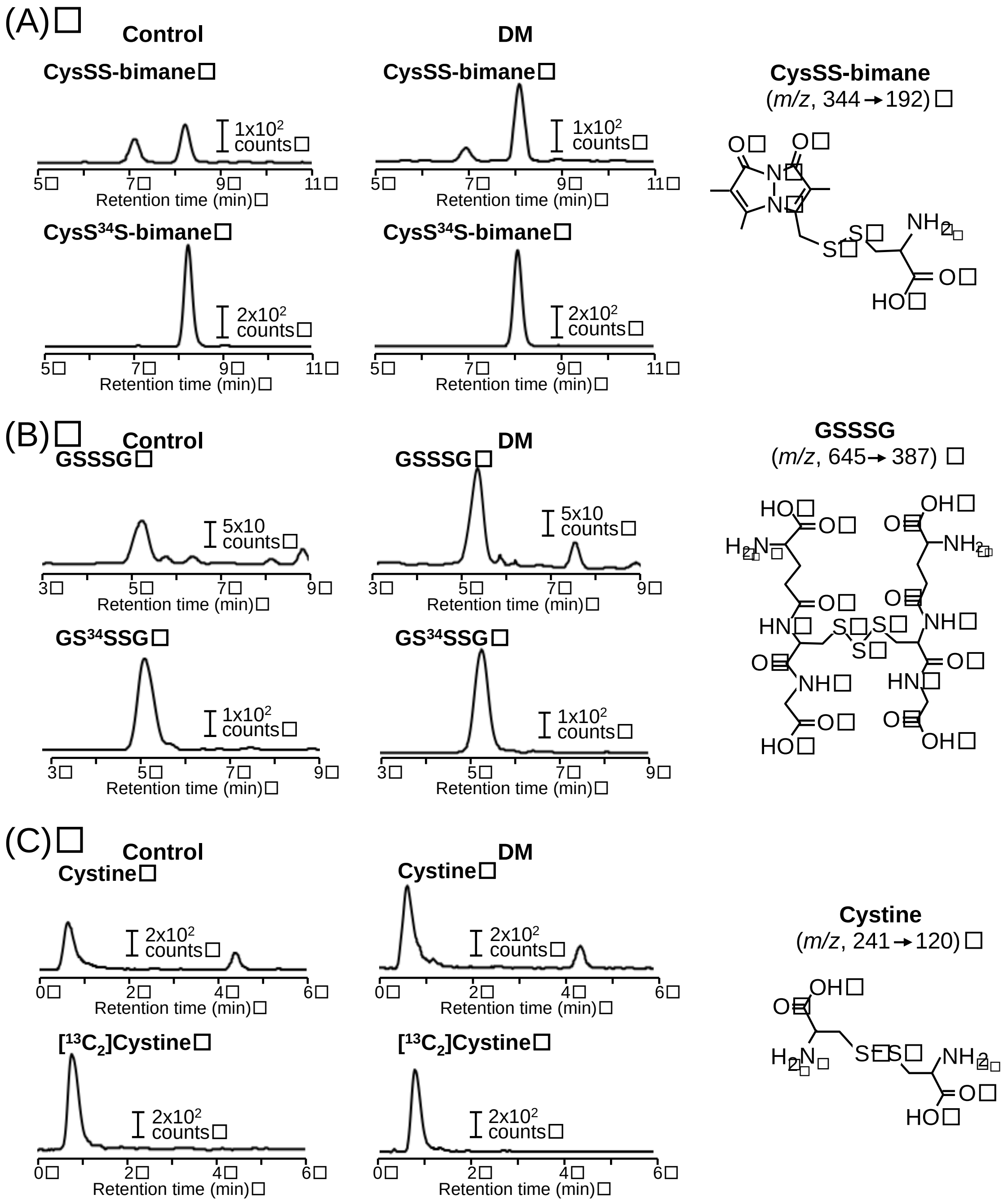

Supplementary Figure S1

Representative LC-ESI-MS/MS chromatograms of various metabolites in the aqueous humor: CysSS-bimane adduct (A), GSSSG (B), and cystine (C), with their MS/MS profiles. Cysteine hydropersulfide (CysSSH) was derivatized with monobromobimane. Upper panels: MS/MS chromatograms corresponding to CysSS-bimane (*m/z* 344 → 192), GSSSG (*m/z* 645 → 387), and cystine (*m/z* 241 → 120); lower panels: stable isotope-labeled internal standards.

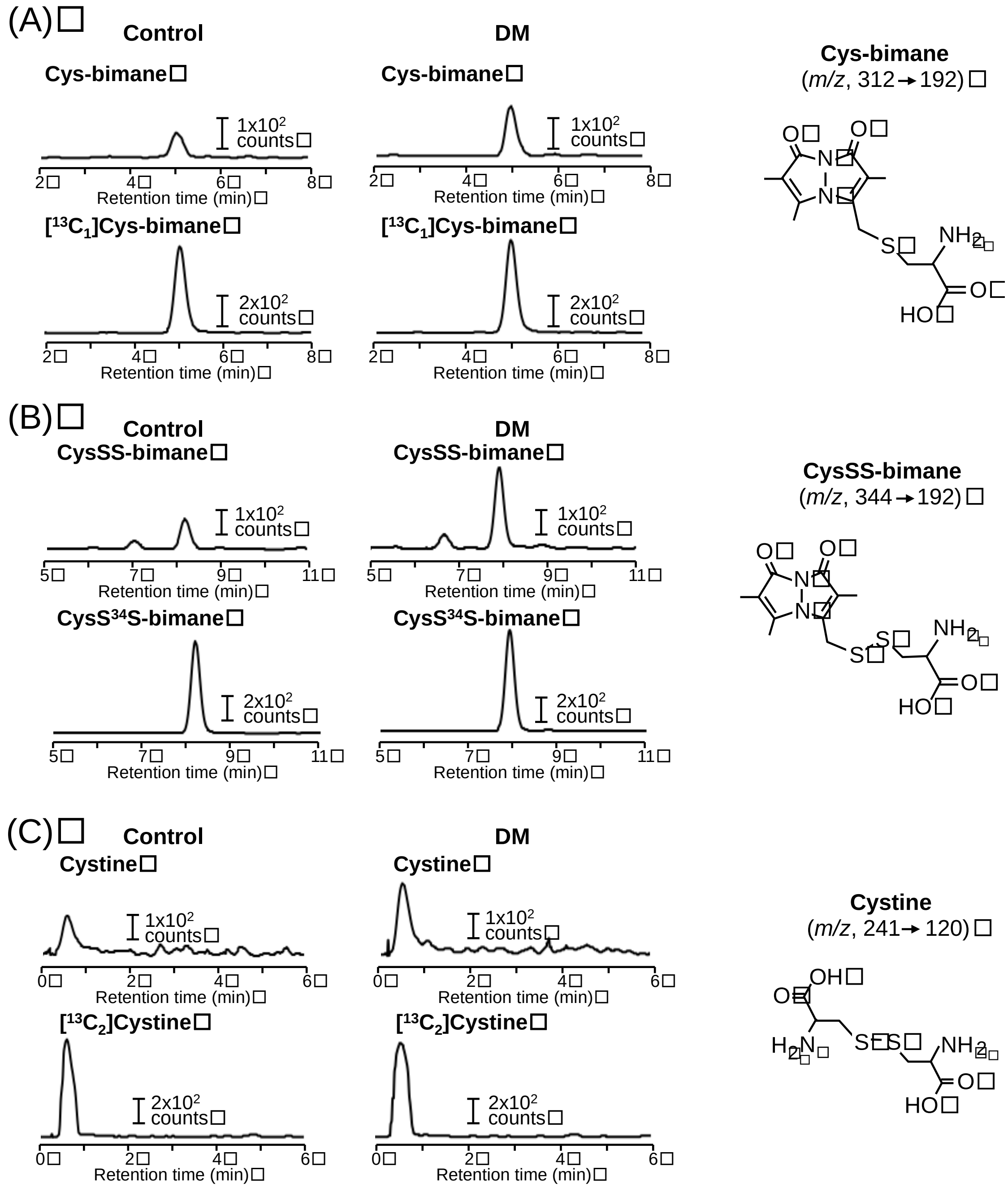

Supplementary Figure S2

Representative LC-ESI-MS/MS chromatograms of various metabolites in the vitreous, including Cys-bimane adducts (A), CysSS-bimane (B), and cystine (C), with their MS/MS profiles. Cysteine (Cys) and cysteine hydropersulfide (CysSSH) were derivatized with monobromobimane. Upper panels: MS/MS chromatograms corresponding to CysS-bimane (*m/z* 312 → 192), CysSS-bimane (*m/z* 344 → 192), and cystine (*m/z* 241 → 120); lower panels: stable isotope-labeled internal standards.

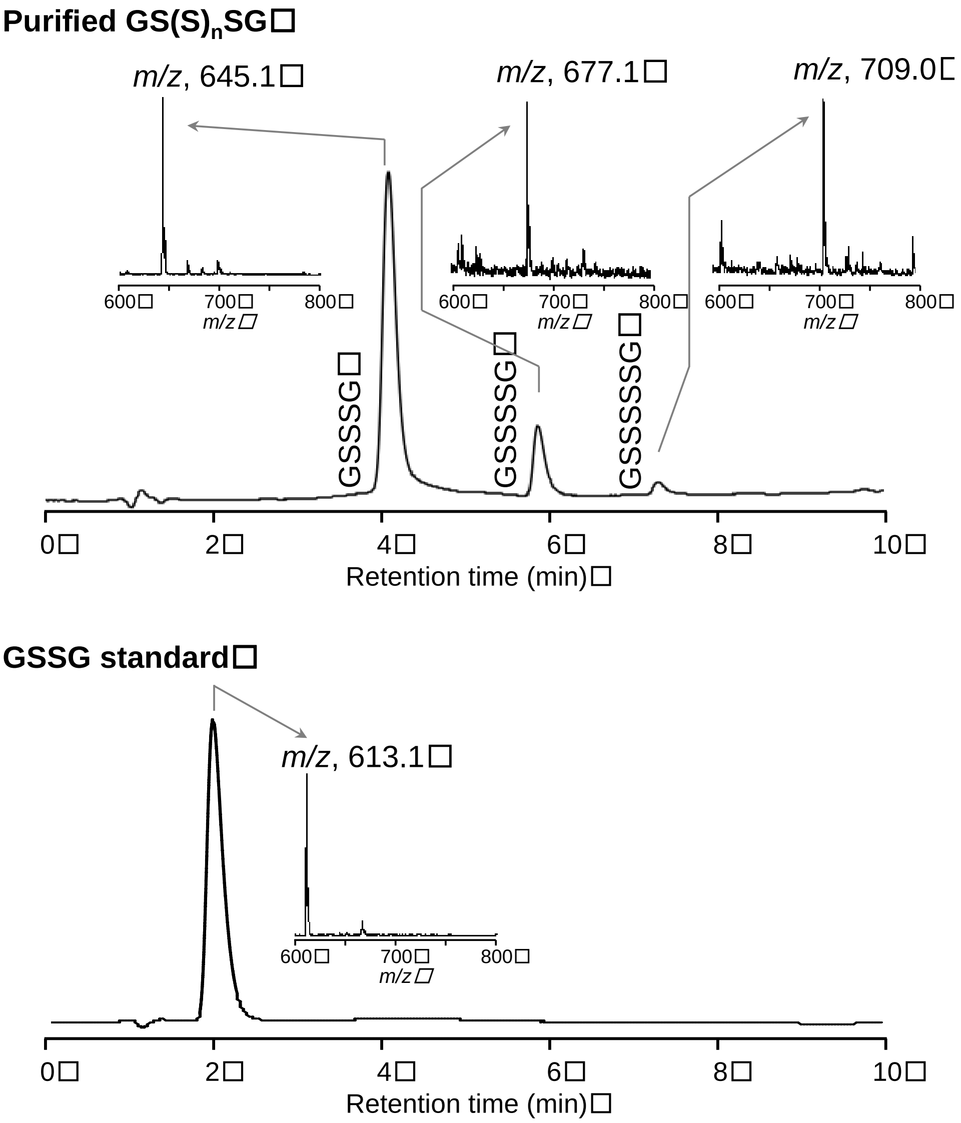

Supplementary Figure S3

HPLC chromatograms of purified GS(S)$_n$SG compounds and their MS spectra. Purified GS(S)$_n$SG (upper panel) and standard GSSG (lower panel) underwent an HPLC analysis (Prominence, Shimadzu Corporation, Kyoto, Japan) with a YMC-Triart C18 column elution (50 × 2.0 mm inner diameter, YMC, Kyoto, Japan), with a linear 3-80% methanol gradient for 15 min in 0.1% formic acid at 40 ºC (flow rate 0.2 ml/min) and peak detection at 254 nm. The insets show GS(S)$_n$SG and GSSG mass spectra obtained with an Agilent 6430 Triple Quadrupole LC-mass spectrometer with a positive scan mode (*m/z*, 600-800). GS(S)$_n$SG includes GSSSG (*m/z*, 645.1), GSSSSG (*m/z*, 677.1), and GSSSSSG (*m/z*, 709.0), but not GSSG (*m/z*, 613.1).

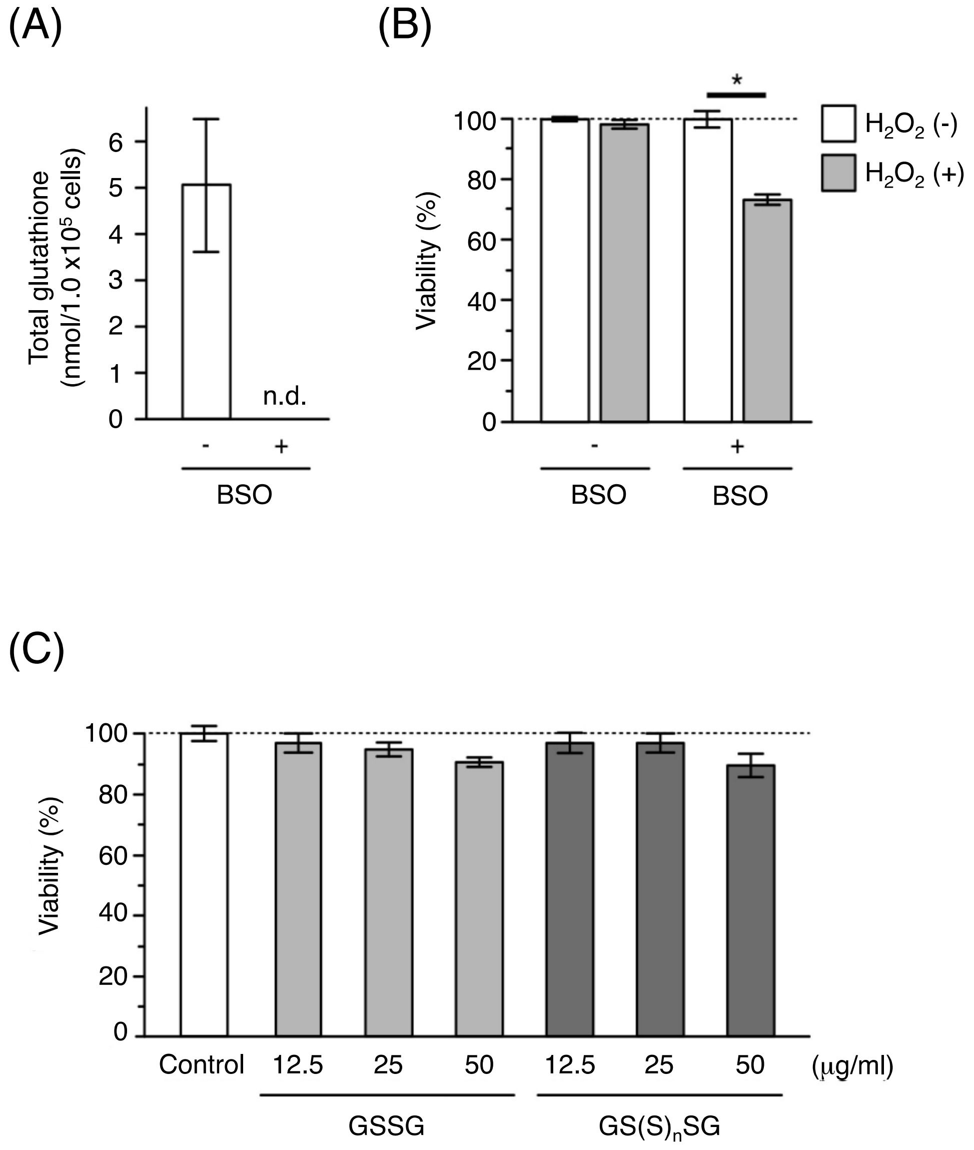

Supplementary Figure S4

Depletion of endogenous glutathiones and the toxicity of GSSG and $GS(S)_nSG$ in RGC-5 cells. (A) Quantification of endogenous glutathiones in RGC-5 cells. The total amount of glutathiones in RGC-5 cells that did or did not undergo treatment with BSO for 18 hours was measured using the GSSG/GSH Quantification Kit (Dojindo Laboratories). Note that the BSO-treated RGC-5 cells had a much lower amount of glutathiones than the untreated cells. Glutathione measurements are the mean of three independent experiments. The error bars indicate the standard error of the mean; nd: not

detected. (B) The effect of glutathione depletion on oxidative stress-induced apoptosis in RGC-5 cells. Glutathione-depleted-RGC-5 and control cells were cultured in a medium containing H_2O_2 (100 µM) for 90 minutes, and cell viability was then evaluated with the Alamar Blue assay. H_2O_2 induced a significant reduction in cell viability in the BSO-treated RGC-5 cells (H_2O_2(-): 100.0 ± 2.9 %; H_2O_2(+): 73.3 ± 1.7 %) but not the BSO-untreated cells (H_2O_2(-): 100.0 ± 0.9 %; H_2O_2(+): 98.0 ± 1.6 %), indicating that glutathione depletion increased the vulnerability of the cells to H_2O_2-induced oxidative stress. (C) The effect of $GS(S)_nSG$ on the survival of BSO-treated RGC-5 cells. Glutathione-depleted RGC-5 cells were incubated with various concentrations of $GS(S)_nSG$ and GSSG in the absence of H_2O_2 to investigate toxicity of these compounds in the cells. No significant decrease in viability was observed in the RGC-5 cells cultured with $GS(S)_nSG$ and GSSG at any concentration, including 25 µg/ml, in comparison with the controls (Dunnett's test). Cell viability in graphs B and C represents the mean of 6 and 4 independent experiments, respectively. The error bars indicate the standard error of the mean. *$P < 0.001$ (Student's t-test). The dotted lines in graphs B and C indicate 100%.

Supplementary Table S1: Sulfide-related metabolite profiling of plasma, aqueous humor, and vitreous in patients with diabetic retinopathy and normal control□

Analyte	□	**Plasma**			□	**Aqueous humor**			□	**Vitreous**		
	□	**Control (*n*=7)**	**DM (*n*=7)**	***P* value**		**Control (*n*=20)**	**DM (*n*=12)**	***P* value**		**Control (*n*=11)**	**DM (*n*=18)**	***P* value**
GSH	(mM)	1.46 ± 0.07	1.46 ± 0.16	0.969		0.74 ± 0.10	0.55 ± 0.11	0.226		0.16 ± 0.06	1.67 ± 0.92	0.225
GSSH	(mM)	0.033 ± 0.01	0.015 ± 0.01	0.316		0.08 ± 0.01	0.10 ± 0.03	0.269		0.07 ± 0.02	0.14 ± 0.04	0.205
Cys	(mM)	3.59 ± 0.62	3.64 ± 1.07	0.969		1.23 ± 0.18	1.28 ± 0.25	0.862		0.28 ± 0.06	0.66 ± 0.14	0.049
CysSSH	(mM)	0.28 ± 0.03	0.27 ± 0.02	0.920		0.14 ± 0.01	0.28 ± 0.09	0.046		0.07 ± 0.01	0.18 ± 0.02	<0.001
GSSG	(mM)	1.98 ± 0.86	1.22 ± 0.16	0.401		0.66 ± 0.30	0.22 ± 0.09	0.280		2.02 ± 0.47	4.97 ± 1.73	0.202
GSSSG	(mM)	0.19 ± 0.04	0.15 ± 0.04	0.516		0.07 ± 0.02	0.19 ± 0.03	0.002		0.15 ± 0.03	0.13 ± 0.03	0.764
Cystine	(mM)	11.67 ± 0.53	11.71 ± 2.08	0.849	□	6.18 ± 0.57	12.58 ± 2.68	0.006	□	2.54 ± 0.41	7.66 ± 0.79	<0.001

Data are means ± s.e.m.
